# Sarcoid-like Lung Disease as a Reaction to Silica from Exposure to Bentonite Cat Litter Complicated by End-Stage Renal Failure—A Case Report

**DOI:** 10.3390/ijerph191912921

**Published:** 2022-10-09

**Authors:** Joanna Hubska, Urszula Shahnazaryan, Marek Rosłon, Benedykt Szczepankiewicz, Kostiantyn Nikiforow, Marcin Pisarek, Małgorzata Barnaś, Urszula Ambroziak

**Affiliations:** 1 Student Scientific Club “Endocrinus” Affiliated to Department of Internal Medicine and Endocrinology, Medical University of Warsaw, 1a Banacha St., 02-097 Warsaw, Poland; 2 Department of Internal Medicine and Endocrinology, Medical University of Warsaw, 1a Banacha St., 02-097 Warsaw, Poland; 3 Department of Pathology, Medical University of Warsaw, 7 Pawińskiego St., 02-106 Warsaw, Poland; 4 Institute of Physical Chemistry Polish Academy of Sciences, Kasprzaka 44/52 Str., 01-224 Warsaw, Poland; 5 Department of Internal Medicine, Pulmonary Diseases and Allergy, Medical University of Warsaw, 1a Banacha St., 02-097 Warsaw, Poland

**Keywords:** sarcoid-like lung disease, bentonite cat litter, silica-related disease, end-stage renal failure

## Abstract

A 44-year-old woman was admitted to hospital with end-stage renal failure, productive cough, and decreased exercise tolerance. She had owned nine cats, which resulted in long-term exposure (18 years) to silica-containing bentonite cat litter. High-resolution computed tomography of the chest showed micronodular lesions in the lungs, and mild mediastinal lymphadenopathy. A lung biopsy revealed multinucleated giant cells, some of which had birefringent material and Schaumann bodies. X-ray photoelectron spectroscopy revealed the presence of silicon in the lung biopsy specimen, as well as in the patient’s cat litter. The pulmonary condition was suggestive of sarcoid-like lung disease, rather than silicosis, sarcoidosis, or hypersensitivity pneumonitis, according to the clinicopathological findings. Renal failure appeared to be a result of chronic hypercalcemia due to extrarenal calcitriol overproduction in activated alveolar macrophages. Ultimately, the patient was diagnosed with sarcoid-like lung disease complicated by end-stage renal failure from exposure to bentonite cat litter. Therapy with steroids, in addition to elimination of the bentonite cat litter exposure, resulted in a significant improvement in the health condition. At a follow-up visit after 4 months, an almost complete resolution of the lung lesions and a significant improvement in renal function were observed.

## 1. Introduction

Exposure to silica dust is one of the oldest known causes of pulmonary diseases, and is associated with a variety of occupations, such as construction, quarrying, and chemical industries [1]. While the inhalation of fine silica particles is a well-recognized risk factor for silicosis, several studies have reported an association between silica exposure and sarcoidosis or sarcoid-like granulomatous lung diseases [2,3,4,5,6,7,8]. According to the literature, silica exposure may also be a trigger for other conditions, including hypersensitivity pneumonitis (HP), lung cancer, tuberculosis, chronic obstructive pulmonary disease, and kidney disease [7,9,10,11]. 

Silica (also known as silicon dioxide, SiO_2_) is a widely abundant mineral compound of the Earth’s crust, and can be found in rocks, concrete, artificial stone, glass, and other industrial materials [11,12]. Silica occurs in non-crystalline (amorphous) and crystalline forms. The amorphous form does not appear to cause clinically significant complications when inhaled; however, the inhalation of crystalline silica may be a trigger for pulmonary disease [1]. To contribute to pulmonary disease, silica particles must be small enough (<5 μm) to reach the distal airways and alveoli. Respirable crystalline silica is colorless, odorless, non-irritating, and does not cause direct health damage; for these reasons, it might go unnoticed in the workplace or any other place in which it is prevalent [11]. Crystalline silica is a component of bentonite, which is a group of natural clays that are used to manufacture pet litter [13]. Bentonite cat litter (BCL) is used worldwide due to its water-absorption qualities and wide availability. 

Nowadays, the burden of silica-associated diseases remains high and treatment options are limited [11]. Moreover, it seems that individuals with no occupational exposure to silica, such as cat owners using BCL, may also be affected. Interestingly, silica-containing cat litter has been associated with susceptibility to sarcoidosis [14]. 

Here, we present an unusual case of sarcoid-like lung disease (SLLD) from exposure to BCL complicated by end-stage renal failure (ESRF). Our objective is to alert clinicians to the possibility of severe health complications in individuals exposed to BCL and to emphasize the importance of detailed patient history-taking.

## 2. Case Presentation

**Figure 1:**
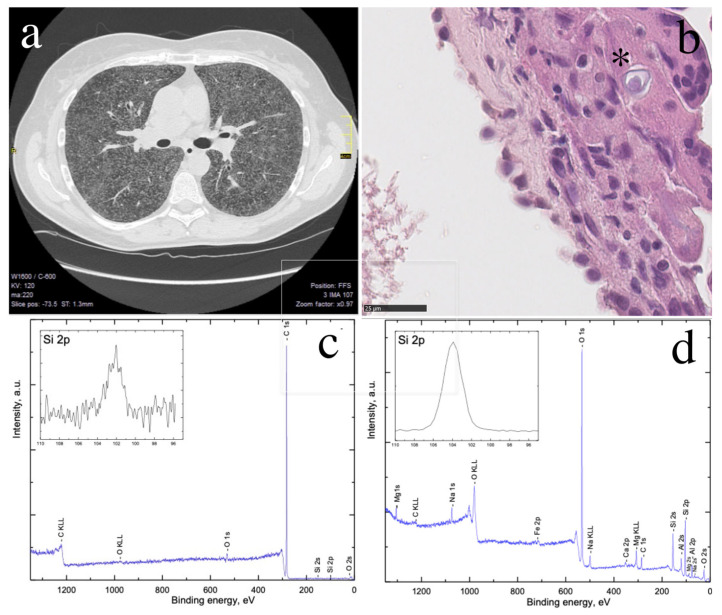
(**a**) High-resolution computed tomography scan with lung windowing obtained on hospitalization. Axial image demonstrates innumerable 1–3 mm diameter nodules randomly distributed throughout both lungs, right paratracheal lymphadenopathy (up to 10 mm), an enlarged lymph node in the aortopulmonary window (13-mm), and small subpleural nodules (up to 3 mm); (**b**) transbronchial lung biopsy microscopic slide (hematoxylin and eosin staining, ×80) showing a giant cell reaction and a Schaumann body (indicated by the black asterisk); (**c**) X-ray photoelectron spectroscopy (XPS) analysis of the lung specimen with peaks for silicon (102.2 eV); (**d**) XPS analysis of cat litter used by the patient with peaks for silica (103.7 eV). The peaks for sodium, iron, calcium, magnesium, and aluminum originating from the cat litter material.

A 44-year-old woman was referred to the hospital for an evaluation of renal failure that had been progressing over the past 5 months (Table 1). The patient reported productive cough, fever (<38 °C), decreased exercise tolerance, and unintentional weight loss (10 kg in 3 weeks). On physical examination, the patient was conscious. Cardiac, pulmonary, abdominal, neurologic, musculoskeletal, and cutaneous examinations were normal. Blood tests showed hypercalcemia, a significant decline in estimated glomerular filtration rate, an increased creatinine level, a decreased parathormone level, and a relatively increased level of calcitriol (Table 1). High-resolution computed tomography (HRCT) of the chest revealed densely patterned micronodular lesions (1–3 mm) scattered throughout the parenchyma of the lungs, with no clear predilection for specific parts of the lungs, and mild mediastinal lymphadenopathy (Figure 1a). In a transbronchial lung biopsy, macroscopically chronic bronchitis was found. Bronchoalveolar lavage fluid (BALF) was cell-rich, with an increased proportion of lymphocytes (37%). Histopathological examination of the lung biopsy specimen showed multinucleated giant cells, some with birefringent material and Schaumann bodies in the cytoplasm (Figure 1b). X-ray photoelectron spectroscopy (XPS) analysis revealed the presence of silicon (Si2p–102.2 eV) in the lung biopsy specimen (Figure 1c). Silicon (Si2p–103.7 eV) was also detected in the patient’s cat litter (Figure 1d).

**Figure 2:**
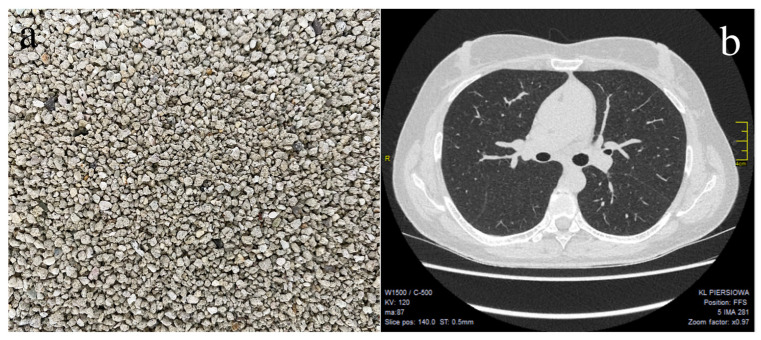
(**a**) Bentonite cat litter used by the patient consistently for 18 years. Granule size: 0.5–2 mm; (**b**) High-resolution computed tomography scan with lung windowing obtained after 4 months with no exposure to bentonite cat litter and completion of steroid therapy. Axial image demonstrates a substantial decrease in micronodular lesions. Right paratracheal lymphadenopathy (up to 10 mm), an enlarged lymph node in the aortopulmonary window (13-mm), and small subpleural nodules (up to 3 mm) were still noted.

A detailed patient history excluded occupational or environmental exposure to crystalline silica; however, she acknowledged having had nine cats at home. In a 44m^2^ apartment, she had nine cat litter boxes, all of which had been filled with BCL (Figure 2a) for 18 years. 

The patient was commenced on prednisone (step-down 30-day therapy: 30 mg/day for 12 days, then 10 mg/day for 12 days, then 5 mg/day for 6 days) and was discharged with a recommendation to completely avoid BCL. At a follow-up visit 4 months later, she reported cough resolution, and a significant improvement in renal function was observed (Table 1). Furthermore, a follow-up HRCT revealed almost complete regression of the micronodular lesions in both lungs (Figure 2b).

## 3. Discussion

We believe that our patient’s disease was a result of the chronic inhalation of bentonite dust, as we excluded other possible causes and observed a significant improvement after the causative agent had been removed. The presence of silicon in the patient’s biopsy specimen, which was confirmed by XPS analysis, supported the association between silica exposure and the patient’s lung disease. Furthermore, steroid therapy in addition to eliminating exposure to the causative agent resulted in a significant improvement in the health condition. At a follow-up visit after 4 months, an almost complete resolution of the lung lesions and a significant improvement in renal function were observed.

Differential diagnosis of the pulmonary manifestations included chronic silicosis, sarcoidosis, and HP, but the clinicopathological features were not entirely specific for any of the diseases. The renal failure appeared to be an indirect result of hypercalcemia caused by extrarenal calcitriol overproduction. Ultimately, a diagnosis of SLLD from exposure to BCL complicated by ESRF was made.

Silicosis is a lung disease resulting from the inhalation of respirable crystalline silica dust. The general pathological mechanisms of silicosis involves activated alveolar macrophage (AM) cytotoxicity, with effects of silica dust leading to fibrosis and the formation of pathognomonic silicotic nodules [5,15]. Chronic silicosis usually develops after more than 10 years of exposure to a low–moderate dose of silica, and occurs in a simple chronic form or as progressive massive fibrosis. Simple chronic silicosis is characterized by small (<1 cm in size) and hard nodules in the upper lung lobes, while progressive massive fibrosis is characterized by conglomerate masses (opacities > 1 cm) which result from the fusion of silicotic nodules [16].

The diagnosis of silicosis can be made after more likely diseases have been excluded, and is based on documented exposure to silica dust and radiographic findings evaluated according to The International Labor Organization’s International Classification of Radiographs of Pneumoconiosis [15]. Given the 18-year exposure to silica-containing litter, the clinical findings, and the confirmed presence of silicon in the lung tissue, we believe that the involvement of silicosis in the overall diagnosis was firmly established. However, the rapid improvement of the lung lesions during steroid therapy goes against a diagnosis of silicosis, as this is not typically indicative of pneumoconiosis. Indeed, such a response to steroid treatment may occur in sarcoidosis, whereas silicosis results in irreversible lung damage [17,18]. Moreover, no silicotic nodules or signs of fibrosis were found in the patient’s biopsy specimen, and the micronodular shadow on the chest CT was not typical of silicosis caused by 18 years of exposure to silica. 

Sarcoidosis is a systemic disease of unclear etiology and is diagnosed based on three major criteria. These criteria are: clinical presentation compatible with sarcoidosis, the presence of non-necrotizing granulomatous inflammation in one or more tissue samples, and the exclusion of alternative causes of granulomatous disease [19]. Various causative agents have been linked to sarcoidosis, including silica-containing cat litter [7,14]. Many features were consistent with sarcoidosis in the current case, mainly the calcitriol meditated hypercalcemia and the good response to steroid therapy. On the other hand, no classic sarcoid granulomas were found in the lung tissue, and the etiology associated with silica exposure was firmly established. Interestingly, some authors suggest that there may be a link between silicosis and susceptibility to sarcoidosis [5,6,20]. 

HP has clinical, radiological, and histopathological features that overlap with sarcoid disease [8]. For example, the BALF analysis of patients with both HP and sarcoidosis reveals a predominance of lymphocytes that was also seen in our patient. The fact that steroids were remarkably effective, and that the symptoms improved after removal of the causative agent, are also suggestive of HP. On the contrary, calcitriol-mediated hypercalcemia and ESRF are not typically associated with HP; however, they are frequently seen in the course of sarcoid disease.

Sarcoid-like reaction is an immune-mediated response to certain antigens, which relate to localized clinical features and do not fulfill the sarcoidosis criteria [18]. Considering that sarcoid-like reactions have been described in patients with pneumoconiosis, alongside the exposure history and clinicopathological features of a granulomatous disease in our patient, we decided to diagnose SLLD rather than sarcoidosis or HP [3,14,20].

From a clinician’s perspective, an important aspect of silica exposure is its possible complications. Indeed, some of them can be non-characteristic and, therefore, difficult to treat unless the causative agent is correctly identified. It is worth noting that our patient was referred because of ESRF, which was an indirect result of calcium homeostasis disorder, and not due to respiratory symptoms. The renal deterioration was a result of chronic hypercalcemia, which was a consequence of extrarenal calcitriol overproduction in activated AMs, due to the enzyme 1-alpha-hydroxylase. As a result, increased calcium absorption in the small intestine and a suppression of parathormone secretion were observed [21]. Such mechanisms are frequently seen in granulomatous diseases. According to our clinical experience and the literature, in cases of silicosis or sarcoidosis, hypercalcemia rarely results in ESRF before other more severe symptoms occur [9,22]. 

## 4. Conclusions

To conclude, it might be difficult to differentiate between silicosis, sarcoidosis and HP based only on radiological patterns and history of exposure. The significant improvement of our patient’s health condition after limiting exposure to the causative agent emphasizes the importance of a detailed patient history in the diagnostic process and thus, in the choice of appropriate treatment. In cases of isolated calcitriol level increase, an extensive workup for granulomatous and inflammatory diseases, including a thorough history of pulmonary exposures, should be performed to help identify the cause of hypercalcemia. Our patient had an abnormally large and lengthy exposure, but similar health complications may occur, for example, in workers in the zoological industry. Given this case, it would seem reasonable to require BCL manufacturers to place information regarding potential health complications and a recommendation to use protective masks on their packaging.

## Figures and Tables

**Table 1:** Serum levels.

	5 Months Prior to Admission	2 Months Prior to Admission	2 Days Prior to Admission	Day of Admission to Hospital	After 10 Days of Steroid Therapy	4 Months after Discharge from the Hospital
eGFR (ml per minute per 1.73 m^2^) RR > 60	58	36	9	8	22	51
Creatinine (mg per deciliter) RR: 0.4–1.2	1.05	1.55	4.96	6.15	2.6	1.28
Calcium (serum) (mmol per liter) RR: 2.15–2.6	ND	ND	ND	3.81	2.23	2.35
Calcitriol (pg per milliliter) RR: 19.9–79.3	ND	ND	ND	67.3	ND	ND

Abbreviation: ND—no data; RR—reference range, eGFR—estimated glomerular filtration rate.

## Data Availability

The datasets used and/or analyzed during the current study are available from the corresponding author on reasonable request.

## Abbreviations

BCL—bentonite cat litter; SLLD—sarcoid-like lung disease; ESRF—end-stage renal failure; XPS—X-ray photoelectron spectroscopy; HRCT—high-resolution computed tomography; ND—no data; RR—reference range; AM—alveolar macrophage; eGFR—estimated glomerular filtration rate; BALF—Bronchoalveolar lavage fluid; HP—hypersensitivity pneumonitis.
